# Cryptococcosis of lumbar vertebra in a patient with rheumatoid arthritis and scleroderma: case report and literature review

**DOI:** 10.1186/1471-2334-13-128

**Published:** 2013-03-07

**Authors:** Heng-Xing Zhou, Guang-Zhi Ning, Shi-Qing Feng, Hong-Wei Jia, Yang Liu, Hong-Yong Feng, Wen-Dong Ruan

**Affiliations:** 1 Department of Orthopaedics, Tianjin Medical University General Hospital, Anshan Road 154, Heping District, Tianjin, 300052, PR China

**Keywords:** Cryptococcosis, Lumbar vertebra, Rheumatoid arthritis, Scleroderma, Fluconazole

## Abstract

**Background:**

Although cryptococcosis mainly occurs in the central nervous system and lungs in immunocompromised hosts, it can involve any body site or structure. Here we report the first case of primary cryptococcosis of a lumbar vertebra without involvement of the central nervous system or lungs in a relatively immunocompromised individual with rheumatoid arthritis and scleroderma.

**Case presentation:**

A 40-year-old Chinese woman with rheumatoid arthritis diagnosed 1 year beforehand and with a subsequent diagnosis of scleroderma was found to have an isolated cryptococcal infection of the fourth lumbar vertebra. Her main complaints were severe low back and left leg pain. Cryptococcosis was diagnosed by CT-guided needle biopsy and microbiological confirmation; however, serum cryptococcal antigen titer was negative. After 3 months of antifungal therapy with fluconazole the patient developed symptoms and signs of scleroderma, which was confirmed on laboratory tests. After taking fluconazole for 6 months, the progressive destruction of the lumbar vertebral body had halted and the size of an adjacent paravertebral mass had decreased substantially. On discharge symptoms had resolved and at an annual follow-up there was no evidence of recurrence on the basis of symptoms, signs or imaging investigations.

**Conclusion:**

Although cryptococcosis of the lumbar vertebra is extremely rare, it should be considered in the differential diagnosis for patients with lumbar vertebral masses to avoid missed diagnosis, misdiagnosis and diagnostic delay. Early treatment with antifungals proved to be a satisfactory alternative to surgery in this relatively immunocompromised patient. Any residual spinal instability can be treated later, once the infection has resolved.

## Background

Cryptococcus is an encapsulated fungus, which is widespread in soil, rotten food and avian feces, especially pigeon droppings [1]. Cryptococcosis caused by *Cryptococcus neoformans* var *neoformans* (CNVN) is most likely to occur in immunocompromised hosts with cell-mediated immune deficiency, and is generally viewed as an opportunistic infectious disease [2-4], as there are only a handful of case reports of established infection in immunocompetent individuals [5,6]. Compared with CNVN, *Cryptococcus neoformans* var *gattii* (CNVG) is far more pathogenic and may infect immunocompetent hosts, with a recent outbreak attracting public attention [2,7]. CNVN and CNVG are increasingly thought to be separate species [8]. It is believed that cryptococcosis occurs upon inhalation of fungal propagules, which can then spread to involve any body site or structure through the blood or lymphatic system [3,6]. The central nervous system (CNS) and lungs are the sites most commonly affected, while the skeletal system is involved in 5-10% of all disseminated *Cryptococcus* infections [9-12]. Here, we present a case of primary cryptococcosis involving the fourth lumbar vertebra without respiratory or CNS involvement in a relatively immunocompromised individual with rheumatoid arthritis (RA) and scleroderma.

## Case presentation

A 40-year-old Chinese woman presented to our hospital complaining of low back pain that had been radiating to the left leg for the past 2 months. She reported significant weight loss of 7 kg, but no fever, chills, night sweats, productive cough, headache, dizziness, nausea or vomiting. One year earlier, she had been diagnosed with RA and took oral non-steroidal anti-inflammatory drugs in the first 3 months after the diagnosis. She had no history of tuberculosis or exposure to patients with tuberculosis. There was also no history of trauma to the back or leg and no loss of bowel or bladder function. She worked as a housewife and kept pigeons as pets, which she fed daily.

On admission, the patient was in considerable discomfort. She was apyrexial. Cardiac and abdominal examinations were normal, but on examination of the chest inspiratory crepitus was detected in the lower right lung base. Mild tenderness was found in the back around the L4 area and movement of the spine was restricted. Motor examination was normal, with normal power in both legs, but tactile hypoesthesia of the right medial femoral condyle was evident. The straight leg raising test (SLRT) elicited pain at 70° on the left and was pain free to 90° on the right. The right patellar reflex was normal, but it was diminished on the left. There were no skin nodules.

**Figure 1:**
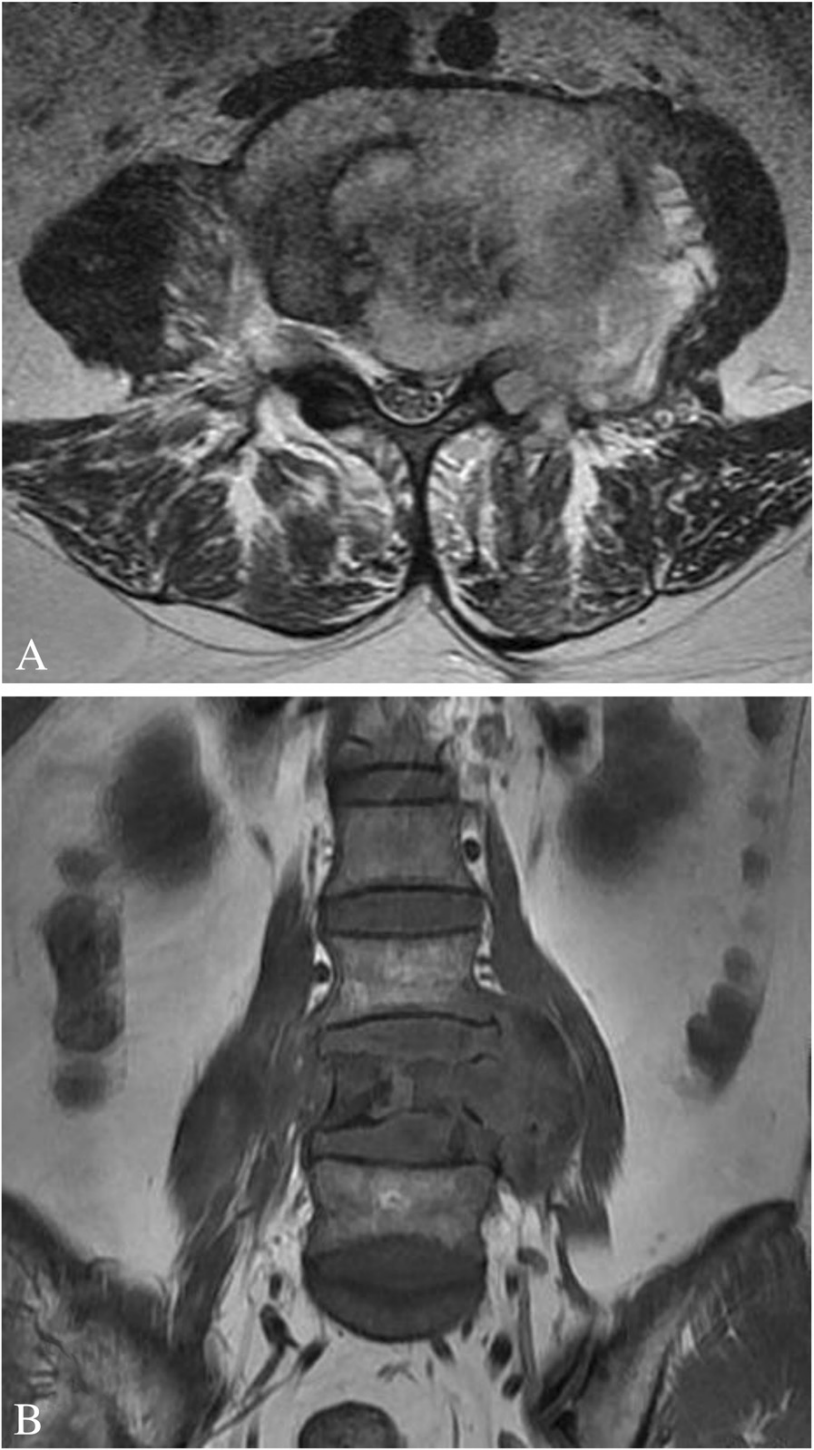
**MRI of the lumbar vertebrae.** (**A**) Transverse section imaging of L4 showed the involvement of left pedicle and transverse process, the paravertebral soft tissue mass and the spinal canal stenosis. (**B**) Coronal section imaging of lumbar vertebrae showed the osteolytic lesions of vertebral body at L4 level.

**Figure 2:**
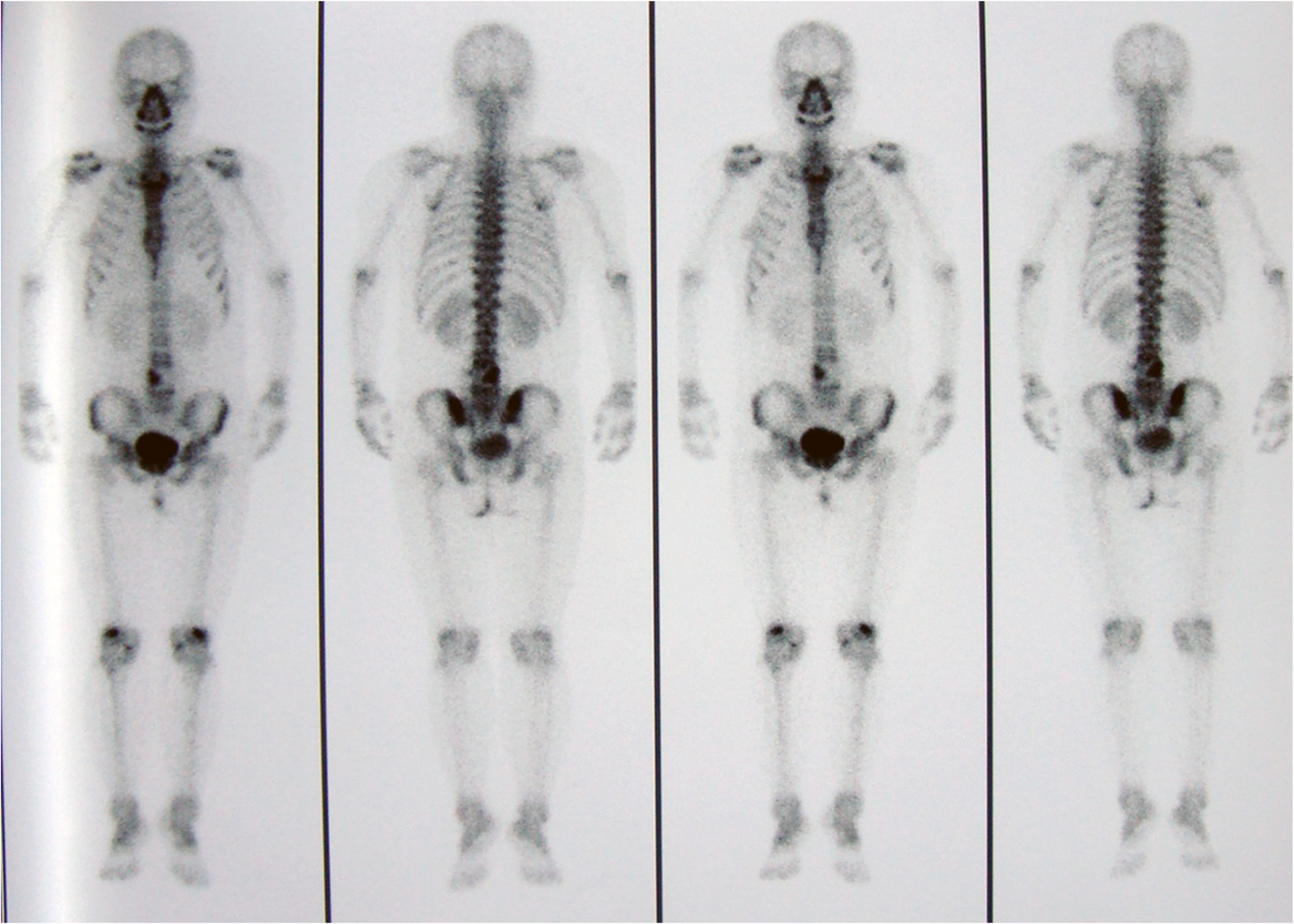
**SPECT examinations.** Increased uptake of technetium-99m in the area of L4 vertebrae.

Magnetic resonance imaging (MRI) undertaken in August 2010 demonstrated osteolytic lesions of the fourth lumbar vertebral body (L4) that involved the left pedicle and transverse process causing secondary spinal canal stenosis at that level (Figure 1A, 1B). Moreover, a paravertebral soft tissue mass was seen on both sides that was larger on the left. The intervertebral discs were relatively preserved. Single photon emission-computed tomography (SPECT) showed increased uptake of technetium-99 m in the L4 vertebra (Figure 2). Routine blood tests revealed: a white blood cell count of 6500/μL with 73.1% neutrophils (normal range: 50-70%), 16.3% lymphocytes (normal: 20-40%), 1.1% eosinophils (normal: 0.5-5%), 0.2% basophils (normal: 0-1%) and 8.5% monocytes (normal: 3-8%); a hemoglobin of 10.7 g/dL (normal: 11.0-16.0 g/dL) and a platelet count of 368,000/μL (normal: 100,000-300,000/μL). The erythrocyte sedimentation rate (ESR) was 22 mm/h (normal: <20 mm/h) and the C-reactive protein (CRP) was 1.45 mg/dL (normal: <0.80 mg/dL). Tumor markers (including alpha fetoprotein, carcinoembryonic antigen, β2-microglobulin, ferroprotein and estradiol) were not elevated, serum cryptococcal antigen and human immunodeficiency virus (HIV) antibodies were negative, but the tuberculin test (PPD skin test) was strongly positive.

**Figure 3:**
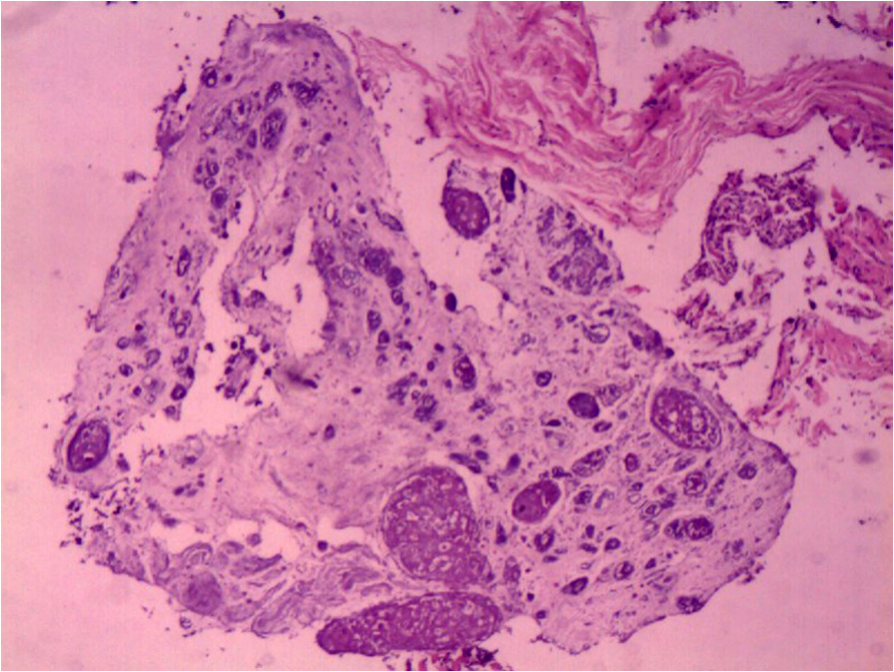
**Histopathological analysis of the paravertebral mass.** Atypical granulomas and massive necrotic tissue with scattered fungal organisms (haematoxylin and eosine staining ×10).

A definitive diagnosis could not be established at this stage. Fourteen days later we performed a CT-guided needle aspiration biopsy and sent the material for culturing of bacteria, *Mycobacteria* and fungi, and also for histopathological analysis. The latter revealed atypical granulomas and large quantities of necrotic tissue with scattered fungal organisms that were identified as *Cryptococcus* by subsequent periodic acid-Schiff (PAS) and Gomori’s methenamine silver (GMS) staining (Figure 3). No malignant cells or epithelioid granulomas were observed in this specimen. Microbial culture revealed the growth of *Cryptococcus* sensitive to fluconazole (FLC) and flucytosine (5-FC), while *Mycobacterium tuberculosis* culture was negative. Because of limited clinical laboratory facilities, we could not conduct tests to distinguish CNVN from CNVG. Considering the antimicrobial sensitivity test results and the side effects of amphotericin B (AMB) and 5-FC, the patient was administered FLC 400 mg/d orally. The patient declined lumbar puncture that would have allowed examination of cerebrospinal fluid.

After 3 months of FLC, the patient complained of pale hands brought on by cold and a sensation of stiffness under the skin in the face and distal extremities: these areas felt indurated on palpation. Laboratory tests found: an elevated γ-globulin (22.3%, normal range: 9.0-16.0%); reduced albumin (57.9%, normal: 60-71%); an immunoglobulin G of 1960 mg/dL (normal: 751-1560mg/mL); a rheumatoid factor (RF) of 29 IU/mL (normal: <29 IU/mL); a circulating immune complex (CIC) of 18 U/mL (normal: <13 U/mL) and an anti-citrulline-containing peptide antibody (anti-CCP) of 20.9 U/mL (normal: <12 U/mL). Also, extractable nuclear antigen anti-Smith surface antigen antibody (ENA anti-SSA antibody), extractable nuclear antigen anti-U1 ribonuleoprotein antibody (ENA anti-U1RNP antibody) and extractable nuclear antigen anti-Scl-70 antibody (ENA anti-Scl-70 antibody) were all positive and antinuclear antibody (ANA) titer was 1:400 (normal: <1:80). ESR had risen to 56 mm/h. As a result, she was diagnosed with scleroderma and was treated with total glucosides of paeony capsules for 16 days. Immunosuppressants were avoided because of concerns about ongoing disseminated infection.

**Figure 4:**
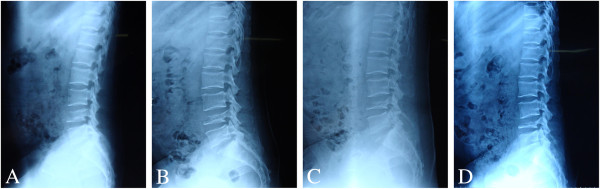
**Plain X-ray of lumbar spine taken every 4 weeks during the treatment with fluconazole.** (**A** and **B**) September and October. (**C** and **D**) No significant changes were found between the imaging taken in November and December.

**Figure 5:**
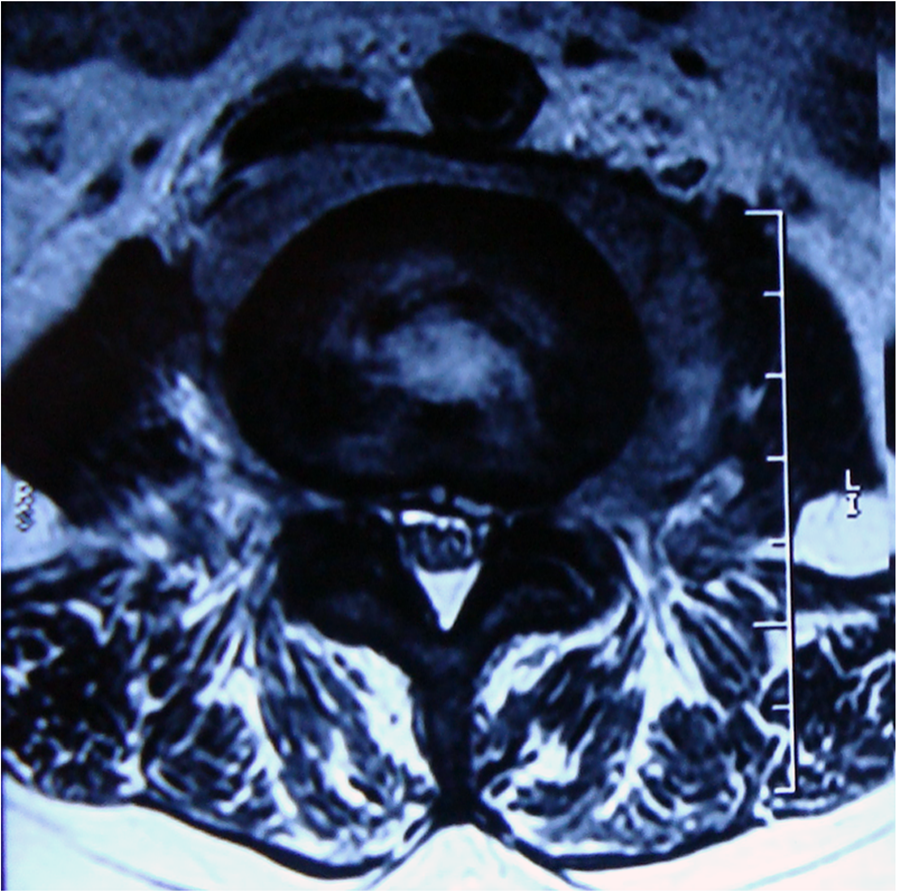
**MRI of the lumbar vertebra taken in November 2010.** The decreased mass and the alleviated spinal cord compression.

During treatment with FLC, a plain X-ray of the lumbar spine was taken every 4 weeks (Figure 4). The X-ray taken in December 2010 (Figure-4D) showed no significant changes when compared with that of the previous month (Figure-4C), suggesting that the destruction of the lumbar vertebral body had abated. Meanwhile, the MRI of November 2010 (Figure 5) showed that the size of the paravertebral mass had decreased when compared with the examination undertaken in August 2011 and the spinal cord compression had been substantially alleviated. After 6 months of continuous oral FLC, her low back and left leg pain had completely resolved. Meanwhile, tactile sensation over the right medial femoral condyle, the SLRT of the left lower limb and the left patellar tendon reflex had recovered to normal. The patient was discharged having made a full recovery and remained asymptomatic and with no radiological evidence of relapse at 12-month follow-up.

## Discussion

Cryptococcosis is rare disease in man and mainly affects the immunocompromised [4]. It is extremely rare in immunocompetent hosts, with an estimated incidence of 0.2 per million per year [13]. The CNS and lungs are most commonly affected and involvement of the musculoskeletal system is uncommon [9]. When bone is affected, the rarest form is a primary infection without involvement of other tissues [12].

On reviewing the English language literature reported since 2000 we found 14 cases (Table 1), including ours, of skeletal cryptococcosis [5,9,14-24]. Among these cases, a total of 28 bony sites and joints were affected, of which 11 were vertebrae, suggesting that vertebral involvement is most common. This finding is consistent with that of John Chleboun [12]. In 1977 he reported a case series of 56 patients in which the vertebrae were involved on 25 occasions out of a total of 117 bony sites and joints. Of the 14 cases, the proportion of men to women was 5:9 and the average age was 37.8 years (range 19-84). Reports published since 2000 do not suggest any predilection for age or gender [14], but this might be a consequence of the small number of reports.

**Table 1:** Main characteristics of 14 cases with skeletal cryptococcosis

**Case no./Reference**	**Age (yr)/sex**	**Underlying diseases**	**Immunosuppressive therapy**	**Bones and joints**	**Treatment**	**Outcome**	**Follow up**
1/[15]	27/F	Lymphocytopenia	None	Left iliac crest, left acetabulum, left femur	Surgery, amphotericin B, fluconazole, itraconazole	Alive (good health)	12 months
2/[18]	24/F	Sarcoidosis	None	T1, T2, T3	Percutaneous puncture drainage, fluconazole, flucytosine, amphotericin B	Alive (no relapse)	16 months
3/[14]	42/M	Diabetes mellitus, renal transplantation	Prednisone, azathioprine, prograf	Left elbow joint, left wrist joint	Sugery, fluconazole	Alive (complete resolution)	6 months
4/[5]	24/F	Tuberculosis	None	T2, T3, left 3^th^ rib	Surgery, fluconazole, amphotericin B	Died 2 weeks after the surgery	None
5/[19]	20/M	Sarcoidosis	Prednisone	T12, L1, L2	Fluconazole	Alive (asymptomatic)	6 months
6/[17]	22/M	None	None	Left 9^th^ rib	Surgery, fluconazole, amphotericin B	Alive (normal)	12 months
7/[20]	19/F	Sarcoidosis	Prednisone	Left humeral head	Fluconazole, amphotericin B	Alive (lesion smaller)	1 month
8/[21]	84/F	Bullous pemphigoid, diabetes mellitus, hypertension, congestive heart failure, hypothyroidism, hypercholesterolemia, atrial fibrillation, degenerative joint disease	Prednisone, azathioprine	Proximal phalanx, intermediate phalanx	Fluconazole, amphotericin B, flucytosine	Died (The cause of death was unknown, and no autopsy was performed.)	None
9/[16]	34/F	None	None	L4, L5	Percutaneous puncture drainage, fluconazole	Alive (full recovery )	None (lost to follow-up)
10/[9]	38/F	Lymphocytopenia	None	Skull vault	Fluconazole	Alive (eradicate the infection)	None (not mentioned)
11/[22]	54/F	None	None	Right frontal bone	Surgery, fluconazole, flucytosine, amphotericin B	Alive (asymptomatic)	6 weeks
12/[24]	38/M	Testicular cancer, sarcoidosis	None	Left clavicle	Surgery, fluconazole	Alive	None (not mentioned)
13/[23]	35/M	Tuberculosis, HIV	None	Left humerus. Left radius, left ulna, left 5^th^ metacarpal	Sugery, fluconazole, amphotericin B	Alive (improved markedly)	19 months
14*	40/F	Rheumatoid arthritis, scleroderma^#^	None	L4	Fluconazole	Alive (no relapse)	12 months

^*^ Present case. ^#^ The scleroderma was diagnosed after the therapy with FLC for 3 months.

In all 14 of the more recent cases, 11 had been diagnosed with other diseases before cryptococcosis (Table 1). Five of the 11 presented with a classical immunodeficiency caused by HIV or immunosuppressant therapies and the remainder had diagnoses consistent with relative immunodeficiency such as sarcoidosis, lymphocytopenia, tuberculosis, diabetes mellitus, renal transplantation, RA and scleroderma [14,25-27]. Thus, complete and relative immunocompromise appear to be the most important risk factors for skeletal cryptococcosis, a view that is consistent with previous reports [5,14,15].

Of the 13 cases reported in the literature, tests to distinguish CNVN from CNVG were not undertaken, despite three cases occurring in the subtropics when CNVG is traditionally viewed as a tropical and subtropical organism [28]. We did not attempt to differentiate between CNVN and CNVG. Cryptococcosis caused by CNVG has never been reported in China, is generally geographically restricted and the patient had no history of travel to areas where CNVG is endemic. Finally, the patient had a history of exposure to pigeon feces, which has been described as a risk factor for CNVN infection rather than CNVG [29,30]. It could be argued that it is important to differentiate between the two organisms because of differences in antifungal susceptibility and the changing ecology and virulence of CNVG [31-33]. We recommend that they should be distinguished if possible to aid diagnosis, direct treatment and improve understanding of the disease.

In our case, the PPD skin test was strongly positive, which suggests active infection with *Mycobacteria*. But the history, clinical symptoms and signs, imaging examinations, mycobacterial culture and histopathological analysis did not support the diagnosis. Hence, lumbar tuberculosis was excluded and we are not able to explain the positive result.

Skeletal cryptococcosis is rare but treatable. Substantial morbidity and mortality can arise from missed diagnosis, misdiagnosis or delay in diagnosis [34]. According to a review of 11 cases of spinal fungal infections, the average delay in diagnosis for 10 of the patients was 99 days and in the last case it took 9 years before the definitive diagnosis was made [35]. One explanation is that the history and radiologic findings resemble a variety of other infectious and neoplastic disorders [36], another is a lack of suspicion of cryptococcosis. Clinicians should be alert to *Cryptococcus* infections and consider appropriate investigations that might contribute to a definitive diagnosis. Cryptococcosis is usually diagnosed by means of a combination of hematological tests, imaging examinations, biopsies and microbial culture. The following should arouse suspicion of the diagnosis. First, although serum cryptococcal antigen is an accurate indicator of cryptococcal infection, it is not positive in all cases [15,16,19,22], just like ours. Second, imaging examinations may lack specificity, resulting in the need for further examinations to differentiate the disease from tuberculosis, neoplastic disorders and yaws, for example. It is noteworthy that the imaging and surgical findings of vertebral cryptococcosis can mimic spinal tuberculosis [5], so biopsies and microbial culture are essential to make the definitive diagnosis.

Of the 14 cases reported since 2000, five were cryptococcosis of the spine (Table 1). All five received antifungal chemotherapy even though there have been cases cured with surgery alone [12]. One of the five was treated with both surgery and antifungal drugs, but the patient died soon afterwards. The cause of death was attributed to the delay in diagnosis and hepatic failure provoked by antitubercular treatment (ATT) [5]. Two of the five cases received at least two types of antifungal drug, while FLC was the sole agent in the other three. Although skeletal cryptococcosis has been most frequently treated with AMB and/or 5-FC [14,17,18], the serious side effects of these drugs led us to prescribe FLC 400 mg/d orally alone for this patient, in compliance the guidelines of the Infectious Diseases Society of America (IDSA) [37]. We were also guided by the sensitivity of the organism grown on culture. Surgery was not offered despite symptomatic spinal cord compression, as we perceived an increased risk of disseminating the infection and at present there are no effective means of preventing this. Moreover, the stress response to surgery might predispose to disseminated infection. We recommend that antifungal agents should be the first line treatment for relatively immunocompromised hosts. Surgery should be considered only if antifungals prove to be ineffective. In this case, surgery to improve the stability of the lumbar spine would be offered at a later date.

## Conclusions

Cryptococcosis of the lumbar vertebra should be considered in the differential diagnosis of patients with lumbar vertebral masses to avoid missed diagnosis, misdiagnosis and diagnostic delay. The successful management of cryptococcosis of the lumbar vertebra with fluconazole alone shows that antimicrobials are a plausible early treatment option that may obviate the need for surgery in relatively immunocompromised patients.

## Consent

Written informed consent was obtained from the patient for publication of this case report and any accompanying images. A copy of the written consent is available for review by the Editor-in-Chief of this journal.

## Abbreviations

CNVN: *Cryptococcus neoformans* var *neoformans*; CNVG: *Cryptococcus neoformans* var *gattii*; CNS: Central nervous system; RA: Rheumatoid arthritis; MRI: Magnetic Resonance Imaging; SPECT: Single photon emission computed tomography; ESR: Erythrocyte sedimentation rate; FLC: Fluconazole; 5-FC: Flucytosine; AMB: Amphotericin B

## Competing interests

All authors have approved the submission and declare that they have no competing interests.

## Authors’ contributions

HXZ, GZN, SQF, HWJ, YL and WDR made substantial contributions to the clinical care. HXZ, GZN, HYF and WDR collected the patient data and involved in the interpretation of data. HXZ and GZN drafted the manuscript. SQF, HWJ and YL revised and edited the manuscript. All authors read and approved the final manuscript.

## Authors’ information

HXZ: PhD Candidate of Orthopaedics. Department of Orthopaedics, Tianjin Medical University General Hospital, Tianjin, PR China. GZN: PhD Candidate and Attending Doctor of Orthopaedics. Department of Orthopaedics, Tianjin Medical University General Hospital, Tianjin, PR China. SQF: PhD and Professor of Orthopaedics. Department of Orthopaedics, Tianjin Medical University General Hospital, Tianjin, PR China. HWJ: Bachelor and Associate professor of Orthopaedics. Department of Orthopaedics, Tianjin Medical University General Hospital, Tianjin, PR China. YL: Master and Attending Doctor of Orthopaedics. Department of Orthopaedics, Tianjin Medical University General Hospital, Tianjin, PR China. HYF: Master of Orthopaedics. Department of Orthopaedics, Tianjin Medical University General Hospital, Tianjin, PR China. WDR: Master and Attending Doctor of Orthopaedics. Department of Orthopaedics, Tianjin Medical University General Hospital, Tianjin, PR China.

## Pre-publication history

The pre-publication history for this paper can be accessed here:

http://www.biomedcentral.com/1471-2334/13/128/prepub
